# Utility of reticulocyte hemoglobin as a new predictor of anemia in intensive care unit patients

**DOI:** 10.3389/fmed.2025.1577047

**Published:** 2025-04-09

**Authors:** Manuel De la Cruz-Garcinuño, Raúl Juárez-Vela, Pablo Lasa-Berasain, Regina Ruiz de Viñaspre-Hernández, Michał Czapla, Lourdes García-Muñoz, Enrique Polo-Andrade, Carmen Sarmiento, Javier Rodero-Martínez, Mirian Alonso-Arias, Saray López-Tornero, Manuel Quintana-Díaz

**Affiliations:** ^1^Doctoral Program in Medicine and Surgery, Autonomous University of Madrid, Madrid, Spain; ^2^Faculty of Health Sciences, GRUPAC Research Group, University of La Rioja, Logroño, Spain; ^3^Research Group on Bleeding Patient Management, IDIPAZ Research Institute, Madrid, Spain; ^4^Intensive Care Medicine Service, University Hospital of Navarra, Pamplona, Spain; ^5^Department of Emergency Medical Service, Faculty of Nursing and Midwifery, Wroclaw Medical University, Wroclaw, Poland; ^6^Intensive Care Medicine Service, La Paz Hospital, Madrid, Spain

**Keywords:** iron deficiency, anemia, intensive care units, erythrocyte indices, ferritins, critical care

## Abstract

**Introduction:**

Iron deficiency and anemia are common complications in critically ill patients, particularly in the Intensive Care Unit setting (ICU), where inflammation and infection are prevalent. Traditional markers like ferritin are unreliable in these contexts due to their behavior as acute-phase reactants. New hematimetric indices, such as Reticulocyte Hemoglobin Equivalent (RET-He) and Delta Hemoglobin Equivalent (Delta-He), may offer better predictive value for anemia in ICU patients.

**Objectives:**

This study aimed to evaluate the predictive utility of RET-He and Delta-He for anemia in critically ill patients and compare their performance with serum ferritin levels.

**Methods:**

A pilot, observational, prospective study was conducted on 40 ICU patients admitted for burns or polytrauma. Hematological and hematimetric parameters were analyzed at admission, 48 h, 4 days, and 7 days post-admission. Mixed-effects regression models were used to assess the predictive value of RET-He, Delta-He, and ferritin for hemoglobin levels and anemia.

**Results:**

Significant reductions in hemoglobin and hematocrit were observed within the first 48 h of ICU admission, while RET-He and Delta-He remained stable. Over 4 and 7 days, decreases in RET-He and Delta-He were strongly associated with lower hemoglobin levels and increased risk of anemia (*p* < 0.01). Ferritin levels did not predict anemia in either period.

**Conclusion:**

RET-He and Delta-He are valuable predictors of anemia in critically ill ICU patients, outperforming ferritin in this context. Their routine use could improve the early detection and management of iron deficiency and anemia in ICU settings.

## 1 Introduction

A deficit in iron levels is critical in patients in the Intensive Care Unit (ICU) due to its role in key biological functions, such as oxygen transport and cellular energy production. Iron deficiency (ID) promotes oxidative stress, exacerbating tissue damage and organ dysfunction. The anemic status caused by iron deficiency worsens the condition of critically ill patients. In septic conditions, multi-organ involvement, or comorbidities, ID anemia is associated with longer ICU stays, higher mortality rates, and organ failure (1).

Laboratory diagnosis of ID is challenging because iron homeostasis is dynamic. No single test provides an accurate assessment of iron absorption, transport, storage, and utilization. Diagnosing ID is especially difficult in patients with acute or chronic inflammatory diseases because most biochemical markers of iron metabolism are affected by the inflammatory process (2).

Serum ferritin (SF) represents most of the iron stored in tissues bound to proteins. Under healthy conditions, there is a direct relationship between reticuloendothelial iron stores and serum ferritin, making it a powerful laboratory test for measuring iron deficiency when no confounding factors are present. However, SF behaves as an acute-phase protein, meaning its levels increase in response to inflammatory and infectious states. In these situations, which are frequent in critically ill patients, normal SF levels cannot exclude ID (3, 4). Other parameters of iron metabolism, such as serum iron, total iron-binding capacity (TIBC), transferrin saturation (TSAT), soluble transferrin receptor (sTfR), zinc protoporphyrin (ZPP), or hepcidin, can support the diagnosis of iron deficiency, but their use is not recommended for diagnosis (5).

Erythrocytes contain approximately half of the body’s iron at any given time. In ID, there is an imbalance between the iron requirements for erythropoiesis and the availability of iron, leading to a reduction in hemoglobin content in reticulocytes and mature erythrocytes. The use of erythrocyte and reticulocyte parameters available in modern flow cytometry-based analyzers is increasingly being included for the detection of early stages of iron-deficiency anemia (6). The reticulocyte hemoglobin equivalent (RET-He) is a parameter reflecting the hemoglobin content in reticulocytes. Since these cells remain in peripheral blood for only 1 to 2 days, RET-He is considered a good indicator of iron availability for erythropoiesis (7) The delta hemoglobin equivalent (Delta-He) estimates the difference between the mean hemoglobin value in reticulocytes and mature red blood cells, indicating the trend of iron incorporation into erythropoietic precursors (8).

Serum ferritin (SF) is the marker commonly used to assess iron levels in the body (3). However, since the introduction of new hematimetric parameters in 2005, several studies have demonstrated their utility in managing iron-deficiency states in patients with chronic kidney disease (5), on hemodialysis (9), with cancer (10), with sepsis (11) or during pregnancy (12). Zuther et al. (8), found that the determination of RET-He and Delta-He was valid for diagnosing iron-deficiency anemia in the ICU (8).

One of the most frequent challenges in Intensive Care is diagnosing iron dysfunction that precedes anemia. There is still a lack of reliable data on the predictive capacity of ID anemia using classical hematological indices and new hematimetric parameters in ICU patients. We consider that, in the ICU context, where inflammation is common, new hematimetric parameters could be a useful tool not only for diagnosis but also for predicting anemia.

The objective of this study was to evaluate the analytical modifications that occur in the first 48 h after admission of trauma patients to the ICU and to measure the capacity of new hematimetric parameters RET-He and Delta-He, in the inflammatory-septic context of ICU patients to predict changes in hemoglobin levels and the onset of anemia within the first 4 and 7 days of admission.

## 2 Methodology

This was a pilot, observational, and prospective study on the ability of hematimetric parameters to identify latent iron deficiency states and predict anemia in critically ill patients. Data were collected in the Polytrauma and Burn Unit, Intensive Care Medicine Service, at the Hospital Universitario La Paz, Madrid, Spain, over a 6-month period following the approval of the ethics committee, with Number HULP: PI-6034.

### 2.1 Inclusion criteria and procedure

Adult patients admitted to critical care units for at least 7 days and who signed informed consent (personally or through a legal representative). Patients with active hematological diseases or those with data recorded in only one of the databases (clinical or hematimetric data) were excluded. Non-probabilistic convenience sampling was used, including data from all patients admitted to the unit who met the selection criteria. Two databases were used for the study. One contained information about the clinical and analytical conditions of the patients. Analytical data were obtained from blood samples routinely collected from ICU patients and analyzed in the hospital’s central laboratory. The second database included data from samples analyzed by the Sysmex® analyzer, which were collected simultaneously with the routine analytical samples. The first day on which the patient had data in both records were designated as “Day 1” and “2 Days” referred to the time point when the patient also had data recorded 2 days after Day 1.

### 2.2 Variables

The following variables were collected:

1. Sociodemographic Variables: Age, sex. Clinical Variables: Reason for ICU admission (burns, sepsis, polytrauma, neurocritical, medical, or others), ICU length of stay, survival (yes/no), ICU admission Sequential Organ Failure Assessment (SOFA) score, with a minimum score of 0 [low organ failure severity] and a maximum score of 24 [high organ failure severity], and Acute Physiology and Chronic Health Evaluation (APACHE) III score, with a minimum score of 0 [low acute illness severity] and a maximum score of 299 [high acute illness severity]; ALI [Acute Lung Injury (yes/no)], Sequential Kidney Injury (SKI) (yes/no), and Extracorporeal Blood Purification Therapy (EBPT) (yes/no), mechanical ventilation (yes/no), days of mechanical ventilation, infection diagnosis (yes/no), infection site (pulmonary, abdominal, bloodstream, urinary, central nervous system, and others), and anemia (hemoglobin value <13 mg/dL).
2. Classical Analytical Indicators: Hemoglobin (Hb), red blood cell count, hematocrit, mean corpuscular volume (MCV), mean corpuscular hemoglobin (MCH), and mean corpuscular hemoglobin concentration (MCHC), ferritin, iron, transferrin, C-reactive protein (CRP), procalcitonin (PCT), transferrin saturation index, and ICIS.
3. Hematimetric Parameters: RET-He and Delta-He, measured through blood count analysis performed on the Sysmex® XN10000 hematology analyzer.

### 2.3 Data analyses

The characteristics of the participants were described using summary statistics. For categorical variables, frequencies and percentages were used. For continuous variables, means (standard deviation) or medians (interquartile ranges) were reported. The Wilcoxon signed-rank test was used to measure changes in hematological parameter values within 48 h after admission. Odds ratios (OR) and 95% confidence intervals (95% CI) were estimated using mixed regression models to predict hemoglobin levels and anemia from serum ferritin, RET-He, and Delta-He. Due to the proportion of missing values in the analysis variables, data imputation was necessary before performing the statistical analysis with mixed-effects models. Without data imputation, the mixed models did not converge. Data imputation was performed using the Random Forest technique, a machine learning method that constructs multiple decision trees to predict missing values by capturing complex, non-linear relationships between variables. In this study, the Random Forest (RF) imputation technique is used due to its ability to model non-linear relationships, handle correlated variables, and flexibly impute categorical and continuous data without requiring strict assumptions.

The following Table 1 illustrates the impact of the imputation process: the left-hand side shows the imputed data, while the right-hand side shows the original data with missing values. This process ensured that data were available for all patients, variables, and time points.

**Table 1:** Mixed-effects models were adjusted for patient-specific random effects and intra-individual correlations over time.

Imputed data							Original data (Without imputation)						
ICU days	Iron	Ferritin	DELTA He	RET He	CRP	HGB	ICU Days	Iron	Ferritin	DELTA He	RET He	CRP	HGB
1	99,48	467,03	−1,94	28,28	43,91	9,05	1	88,36	364,60	−2,74	27,22	44,93	8,76
2	103,91	454,13	1,13	31,93	111,69	12,03	2	95,25	382,67	1,71	32,51	110,42	12,27
3	57,69	451,87	−0,17	30,14	134,74	10,78	3	64,00	386,50	−0,27	29,88	135,00	10,69
4	41,13	443,41	1,45	31,95	147,09	10,57	4	32,71	402,33	1,52	32,17	146,74	10,32
5	52,96	328,10	1,90	32,20	133,47	10,93	5	50,00	75,00	2,35	32,60	134,84	11,08
6	45,13	785,76	−0,21	30,40	165,90	9,91	6	31,00	1,433,00	−0,65	29,99	168,10	9,87
7	50,88	793,54	0,94	31,24	142,06	9,58	7	44,00	734,00	0,92	31,35	143,27	9,62

ICU, intensive care unit; FS, serum ferritin; DELTA-He, delta hemoglobin; RET-He, reticulocyte hemoglobin; CRP, C-reactive protein; HGB, hemoglobin.

## 3 Results

### 3.1 Sample description

During the study period, 74 patients were admitted to the ICU. Of these, 42 patients had records in both databases: the clinical/analytical database and the hematimetry database. These patients were aged between 18 and 84 years, with a mean age of 52 years (SD **±** 8.4). Among them, 23 were men (54.8%) and 19 were women (45.3%). The main reason for admission was burns (40.48%). They had an average ICU stay of 13.5 days and a survival rate of 80.9%. The average severity of their condition, assessed using the APACHE scale, was 11.8, and organ dysfunction, measured by the SOFA scale, was 3.79. Mechanical ventilation was required in 50% of the cases, see Table 2.

**Table 2:** Clinical description of patients on admission.

	Mean (±SD)	*N* (%)
Reason for admission		
Burned		17 (40.48%)
Medical and others		11 (26.19%)
Sepsis		7 (16.67%)
Polytrauma		4 (9.52%)
Neurocritical		3 (7.14%)
Days in UCI	13.48 (±19.47)	
Survival		
No		8 (19.05%)
Yes		34 (80.95%)
Apache score	11.82 (±6.86)	
Sofa score	3.79 (±3.74)	
Acute lung injury		
No		28 (66.67%)
Yes		14 (33.33%)
Mechanical ventilation		
No		21 (50.00%)
Yes		21 (50.00%)
Days on mechanical ventilation	6.17 (±12.38)	
Acute kidney injury		
No		29 (70.73%)
Yes		12 (29.27%)
Thrombosis of extracorporeal device		
No		40 (95.24%)
Yes		2 (4.76%)
Infection		
No		26 (61.90%)
Yes		16 (38.10%)
Hemoglobin	10.97 (±2.67)	
Red blood cell count (RBC)	3.58 (±0.88)	
Mean corpuscular volume (MCV)	94.85 (±6.16)	
Mean corpuscular hemoglobin (MCH)	30.75 (±2.42)	
Mean corpuscular hemoglobin concentration (MCHC)	30.75 (±2.42)	
Hematocrit	33.82 (±8.10)	
Ferritin	792.75 (±539.37)	
Iron	48.20 (±49.24)	
Transferrin	80.00 (±7.39)	
C-reactive protein (CRP)	125.10 (±106.76)	
Procalcitonin	3.98 (±8.83)	
Transferrin saturation index	23.25 (±11.59)	
Immature granulocyte index	2.43 (±2.82)	
Reticulocyte hemoglobin equivalent (pg)	30.62 (±4.09)	
Delta hemoglobin equivalent (pg)	0.30 (±3.72)	

Table 3 shows hematological and hematimetric changes in the first 48 h of ICU admission. Data were obtained at admission and 48 h after admission, revealing that only Hb levels (*p* < 0.01) and hematocrit (*p* = 0.04) decreased significantly. The red blood cell count showed a reduction but did not reach statistical significance (p = <0.01). No significant changes were observed in the other evaluated parameters.

**Table 3:** Hematological and hematimetric changes in the first 48 h.

Parameter	*N*	Initial value median (IQR)	48-hour value median (IQR)	*p*-value
C-reactive protein (CRP)	16	141.8 (64.6–205.3)	146.6 (84.9–209.1)	0.79
Immature granulocyte Index (Immature cell index)	30	2.0 (0.0–3.0)	2.0 (0.3–4.0)	0.14
Reticulocyte hemoglobin equivalent (RET-He)	30	31.5 (26.8–33.7)	31.9 (30.1–34.3)	0.26
Delta hemoglobin equivalent (Delta-He)	30	1.5 (−2.9–3.2)	1.8 (−0.6–3.4)	0.20
Reticulocyte production index	30	1.1 (0.5–1.4)	1.0 (0.3–2.7)	0.66
Hemoglobin	30	10.5 (9.0–12.0)	9.4 (8.4–10.9)	< 0.01
Red blood cell count (10×6 u/L)	30	3.2 (2.8–4.0)	3.0 (2.6–3.2)	0.09
Hematocrit (%)	30	31.95 (27.9–36.4)	29.9 (25.5–34.1)	0.04
Mean corpuscular volume (fL)	30	95.5 (92.6–98.1)	95.1 (91.4–100.9)	0.73
Mean corpuscular hemoglobin (pg)	30	30.7 (29.8–32.5)	30,8 (29.7–31.9)	0.89
Mean corpuscular hemoglobin concentration (g/dL)	30	32.4 (31.2–33.4)	32.1 (31.2–33.3)	0.43
Reticulocyte count (10×6 u/L)	30	0.08 (0.05; 0.12)	0.07 (0.06; 0.11)	0.95
Procalcitonin	30	0.2 (0.1–0.7)	0.2 (0.1–0.8)	0.46

The mean hemoglobin values were not associated with SF level during the first 4 days (*p* = 0.949). However, when the evaluation period was extended to the first 7 days, an inversely proportional and statistically significant relationship was observed between ferritin and hemoglobin levels. Specifically, for each 1-unit increase in ferritin, hemoglobin decreased by 0.0013 units (*p* < 0.01).

Regarding the mean values of the RET-He and Delta-He indices, in both periods, a directly proportional and significant relationship was observed with the mean hemoglobin values. During the first 4 days, for each 1-unit increase in RET-He, hemoglobin increased by 0.2033 units, and by 0.1861 units during the first 7 days (*p* < 0.01). Similarly, for each 1-unit increase in Delta-He, hemoglobin increased by 0.2051 units and 0.2651 units for the 4-day and 7-day periods, respectively (*p* < 0.01). See Table 4.

**Table 4:** Predictive factors for hemoglobin decrease over a period of 4 and 7 days.

	(First 4 days)			(First 7 days)		
Predictors*	Estimates	95% CI	*p*-value	Estimates	95% CI	*p*-value
Ferritin	0.0001	(−0.0015; 0.0016)	0.95	- 0.0013	(−0.0018; −0.0008)	< 0.01
RET-He	0.2033	(0.1291–0.2775)	< 0.01	0.1861	(0.1341; 0.2382)	< 0.01
Delta-He	0.2051	(0.1187; 0.2915)	< 0.01	0.2651	(0.2067; 0.3235)	< 0.01

*Estimates adjusted for UCI days.

Table 5 shows the results estimating the probability of anemia based on SF, RET-He, and Delta-He values, adjusted for days of hospitalization, during the first 4 and 7 days of admission.

**Table 5:** Mixed regression analysis to predict anemia.

	(First four days)			(First seven days)		
Predictors*	OR-a	95% CI	*p*-value	OR-a	95% CI	*p*-value
SF	0.99	(0.99; 1.00)	0.83	1.00	(0.99;1.0)	0.26
RET-He	0.20	(0.13; 0.28)	< 0.01	0.77	(0.69; 0.87)	< 0.01
Delta-He	0.63	(0.51; 0.78)	< 0.01	0.69	(0.60–0.80)	< 0.01

*Odds ratio adjusted for UCI days.

Ferritin values do not predict the occurrence of anemia in either of the two periods. However, a decrease of 1 unit in RET-He values significantly increases the average probability of anemia by 5 times (1/0.20) during the first four days and by 1.3 times (1/0.77) over a longer period of 7 days (*p* < 0.01).

Similarly, a decrease of 1 unit in Delta-He values increases the average probability of anemia by 1.6 times (1/0.63) during the first four days and by 1.5 times (1/0.69) over the 7-day period (*p* < 0.0001).

The relationship between the mean RET-He and Delta-He values and mean SF levels was also measured during the first 4 and 7 days.

During the first four days, RET-He values increased by 0.0021 units for each unit increase in SF However, this slight increase did not reach statistical significance [0.0021 (95% CI: −0.0003; 0.0044), *p* < 0.08]. In contrast, over the 7-day period, the relationship found was inversely proportional. RET-He decreased by a clinically and statistically significant average of 42 units for each additional unit of SF [−42 (95% CI: −54; −31), *p* < 0.00001].

Regarding the relationship between Delta-He and SF during the first four days, the relationship was directly proportional. Delta-He showed a slight but statistically significant increase of 0.0026 units for each additional unit of ferritin [0.0026 (95% CI: 0.0006; 0.0047), *p* < 0.0118]. However, during the seven-day period, the relationship became inversely proportional and significant. Delta-He decreased by an average of 0.003 units for each additional unit of SF [−0.0030 (95% CI: −0.0039; −0.0020), *p* < 0.0001].

To contextualize the comparison of RET-He and Delta-He with other relevant markers, in order to better understand their predictive value in intensive care unit patients, Table 6 summarizes the following markers.

**Table 6:** Summary of the relationship between RET-He and Delta-He.

Marker	Relationship with RET-He	Relationship with Delta-He	Interpretation/Predictive value
Hemoglobin (Hb)	RET-He showed a significant correlation with Hb decrease at 4 and 7 days (p < 0.01).	Delta-He was also significantly associated with Hb decrease at 4 and 7 days (*p* < 0.01).	Both markers predict changes in Hb, reflecting the effectiveness of erythropoiesis and iron availability.
Hematocrit (Hct)	Changes in RET-He were not directly related to Hct within the first 48 h (*p* = 0.26).	Delta-He could correlate with Hct changes due to its relationship with Hb production.	Hct reflects total red blood cell volume, but RET-He and Delta-He provide more specific information on erythropoiesis.
Red blood cell count (RBC)	RET-He did not show significant changes with RBC within the first 48 h (*p* = 0.09).	Delta-He could correlate with RBC in contexts of recent erythropoietic regeneration.	RET-He and Delta-He complement RBC information by evaluating the quality of the produced red blood cells.
Reticulocyte production index (RPI)	RET-He showed stability in relation to RPI (*p* = 0.66), indicating consistent erythropoiesis.	Delta-He might correlate with increases in RPI in cases of active regeneration.	Both markers are useful for evaluating real-time erythropoietic responses.
Mean corpuscular volume (MCV)	RET-He did not show a significant correlation with MCV (*p* = 0.73).	Delta-He also showed no direct correlation with MCV.	MCV measures the average size of red blood cells but does not provide information on the quality of hemoglobin produced.
Mean corpuscular hemoglobin (MCH)	RET-He remained stable in relation to MCH (*p* = 0.89).	Delta-He showed no direct relationship with MCH.	MCH reflects the average Hb content per red blood cell, but RET-He and Delta-He are more sensitive to recent changes.
Mean corpuscular hemoglobin concentration (MCHC)	RET-He did not show a significant correlation with MCHC (*p* = 0.43).	Delta-He also showed no direct relationship with MCHC.	MCHC measures Hb concentration in red blood cells but does not evaluate erythropoietic dynamics.
Procalcitonin (PCT)	RET-He did not show a significant correlation with PCT (*p* = 0.46).	Delta-He also showed no direct relationship with PCT.	PCT reflects inflammation or infection but is not directly related to erythropoiesis or iron metabolism.
Ferritin	RET-He showed a significant association with Hb changes at 7 days, influenced by elevated ferritin (p < 0.01).	Delta-He showed a significant association with Hb changes at 7 days, influenced by elevated ferritin (*p* < 0.01).	Elevated ferritin may reflect inflammation, affecting erythropoiesis; RET-He and Delta-He are useful for assessing functionality.

**RET-He and Delta-He** are dynamic markers that provide more specific information about erythropoiesis and iron availability compared to traditional markers (Hb, Hct, RBC,) **Delta-He** appears to be more sensitive in detecting recent deficits in hemoglobin production, while **RET-He** more generally reflects iron availability. Markers such as **MCV**, **MCH**, and **MCHC** are useful for assessing general red blood cell characteristics but do not provide real-time information on erythropoietic functionality as RET-He and Delta-He do. In the context of inflammation (reflected by elevated ferritin), **RET-He** and **Delta-He** are valuable tools for evaluating the effectiveness of erythropoiesis and the response to iron supplementation.

## 4 Discussion

The aim of this study was to evaluate the changes observed in the blood tests of patients admitted to an ICU for burns and polytrauma and to assess the ability of new hematimetric parameters (RET-He and DELTA-He) to predict hemoglobin decline and anemia secondary to iron deficiency anemia (IDA).

The data from this study show that during the first 48 h of ICU admission, significant reductions in both hemoglobin and hematocrit occur without changes in the hematimetric parameters studied (RET-He and Delta-He). Over a longer period of 4 and 7 days, the measurement of hematimetric parameters emerges as a predictive factor for changes in hemoglobin levels and the onset of anemia.

The decline in hemoglobin and hematocrit values during the first 48 h of ICU admission observed in this study reinforces evidence of the high risk of critically ill patients developing hospital-acquired anemia (13). More than 75% of patients admitted to the ICU for more than 7 days develop anemia (14, 15), which reduces their chances of survival or achieving full functional recovery at 3- and 12-months post-discharge among survivors of severe illnesses (15, 16).

During the first 48 h, the significant drop in hemoglobin and hematocrit values is likely due to hemorrhage from associated injuries such as splenic rupture, fractures, and severe tissue damage. Other contributing factors include frequent blood draws, fluid replacement therapy, and hemolysis. In Warner et al.’s study (2020) (15), 56% of patients developed anemia within 24 h of ICU admission, and 80% had anemia by the time of ICU discharge. Among survivors, higher hemoglobin concentrations at discharge were associated with lower subsequent mortality.

During these initial 48 h, according to this study’s data, RET-He and Delta-He levels do not appear to undergo significant changes, suggesting the maintenance of erythropoietic processes aimed at compensating for red blood cell loss. This finding aligns with current knowledge about the relationship between iron metabolism and hematopoiesis. In this early stage, the body’s iron reserves are mobilized to sustain the synthesis of erythroid progenitors of reticulocytes and erythrocytes (17).

Stimulated erythropoiesis in response to red blood cell loss rapidly consumes available iron, as hemoglobin synthesis in erythroblasts requires large amounts of iron. The restriction of heme and hemoglobin production under iron-deficient conditions could be detected early using new cytometric techniques, even before iron deficiency impairs erythroblast maturation, reduces red blood cell production, or affects mean corpuscular volume and mean corpuscular hemoglobin (18).

According to this study, both RET-He and DELTA-He are predictive indices of changes in hemoglobin concentration and anemia over a 4-day or 7-day period post-admission. However, SF levels in the studied patients do not predict changes in hemoglobin or aid in detecting anemia. These findings support data from other studies confirming the suitability of hematimetric parameters for diagnosing early stages of iron deficiency in both sick and healthy populations (19).

There is consensus among intensivists on the need to treat iron deficiency before it causes anemia, as this condition poses a serious risk to critically ill patients and delays their recovery (11). However, interpreting results from traditional hematological tests can sometimes be challenging, as they are altered by inflammatory or infectious processes, and their normal ranges may not apply. Consequently, many cases of iron deficiency without anemia may go undiagnosed and untreated (20). As shown in this study, during a 7-day period, increased SF levels are associated with a decrease in RET-He and Delta-He values, further highlighting SF limited utility in diagnosing iron deficiency in critically ill patients.

## 5 Implications for practice, variability, and cost-effectiveness

According to Danielson et al. (21), the implementation of hematimetric parameters RET-He and Delta-He in ICU settings represents a significant opportunity to optimize the early detection and management of anemia, as well as to efficiently monitor inflammation and iron status in critically ill patients. In particular, the analysis of Delta-He has been shown to be comparable to IL-6 in predicting mortality, with the advantage of significantly lower costs and the ability to be easily integrated into routine analyses, such as the complete blood count (CBC).

Recent advancements in automated hematological analysis systems have enabled the measurement of various hematimetric indices, such as reticulocyte hemoglobin (RET-He) and erythrocyte hemoglobin (RBC-He), using the same sample collected for a complete blood count. Incorporating these novel hematimetric markers into routine ICU practice, alongside traditional hematological parameters, has the potential to improve the management of iron deficiency in critically ill patients. This is particularly relevant given the limitations and inefficacy of traditional anemia parameters in these settings.

From an economic perspective, Hönemann et al. (22) highlight that the measurement of RET-He and Delta-He is significantly more accessible compared to other traditional biomarkers of iron and inflammation. For instance, the cost of measuring RET-He ranges between €0.70 and €1.00, whereas traditional parameters, such as ferritin (€17.76) and transferrin saturation (€6.71), represent much higher expenses. This positions RET-He and Delta-He as cost-effective and practical alternatives for implementation in intensive care settings.

In this context, the design of a new algorithm that integrates these innovative indicators alongside traditional parameters could significantly improve the diagnosis and treatment of anemia in critically ill patients, maximizing available resources and overcoming the limitations of conventional methods.

### 5.1 Strengths and limitations

This pilot study aimed to assess the utility of novel hematimetric parameters (RET-He and Delta-He) in the early diagnosis of iron deficiency states among critically ill patients. While the sample was not selected using a method that allows for generalization to the broader ICU population, the findings provide valuable preliminary insights. Additionally, the use of a uniform hemoglobin threshold to define anemia for both men and women may have overlooked sex-specific physiological differences, highlighting an area for further investigation.

During the follow-up of 42 patients, 19.05% succumbed to their illnesses, and 4.76% (2 patients) were lost to follow-up. It is important to note that the small sample size limits the ability to draw generalized conclusions, underscoring the need for studies with larger cohorts to validate these results. Furthermore, the sample was drawn from a single, specific Intensive Care Unit, which ensured a consistent clinical environment but may limit the applicability of the findings to other ICUs. Differences in clinical conditions and patient characteristics across ICUs should be considered when interpreting these results such as nutritional status, blood loss, or transfusion practices.

The study employed the Random Forest (RF) technique for data imputation, a sophisticated method capable of capturing complex, non-linear relationships between variables. However, the precision of RF imputation depends on the observed variables, which may introduce biases if the missing data are not random (MNAR). Additionally, the use of RF in small samples may increase variability in the analyses. Despite these limitations, the application of RF reflects a modern and innovative approach to addressing missing data, which can be further refined and validated in future research with larger and more diverse datasets.

## 6 Conclusion

The data support the hypothesis that, in these 40 ICU patients, ferritin levels do not predict the risk of anemia, whereas a decrease in RET-He and Delta-He indices is associated with lower hemoglobin levels and could be clinically useful for predicting IDA.

## Data Availability

The raw data supporting the conclusions of this article will be made available by the authors upon request to author MQ-D, due to legal restrictions on personal data.

## References

[1] Zhang X Holbein B Zhou J Lehmann C. Iron metabolism in the recovery phase of critical illness with a focus on Sepsis. Int J Mol Sci. (2024) 25:7004. doi: 10.3390/ijms25137004, PMID: 39000113 PMC11241301

[2] Fletcher A Forbes A Svenson N Wayne TD. Guideline for the laboratory diagnosis of iron deficiency in adults (excluding pregnancy) and children. Br J Haematol. (2022) 196:523–9. doi: 10.1111/bjh.17900, PMID: 34693519

[3] Peyrin-Biroulet L Williet N Cacoub P. Guidelines on the diagnosis and treatment of iron deficiency across indications: a systematic review. Am J Clin Nutr. (2015) 102:1585–94. doi: 10.3945/ajcn.114.103366, PMID: 26561626

[4] Garcia-Casal MN Pasricha SR Martinez RX Lopez-Perez L Peña-Rosas JP. Serum or plasma ferritin concentration as an index of iron deficiency and overload. Cochrane Database Syst Rev. (2021) 2021:CD011817. doi: 10.1002/14651858.CD011817.pub2, PMID: 34028001 PMC8142307

[5] Padhi S Glen J Pordes BAJ Thomas ME. Management of anaemia in chronic kidney disease: summary of updated NICE guidance. BMJ. (2015) 350:h2258–8. doi: 10.1136/bmj.h2258, PMID: 26044132

[6] Urrechaga E Borque L Escanero JF. Erythrocyte and reticulocyte indices in the assessment of erythropoiesis activity and iron availability. Int J Lab Hematol. (2013) 35:144–9. doi: 10.1111/ijlh.12013, PMID: 23033935

[7] Almashjary MN Barefah AS Bahashwan S Ashankyty I ElFayoumi R Alzahrani M. Reticulocyte hemoglobin-equivalent potentially detects, diagnoses and discriminates between stages of Iron deficiency with high sensitivity and specificity. J Clin Med. (2022) 11:5675. doi: 10.3390/jcm11195675, PMID: 36233545 PMC9572493

[8] Zuther M Rübsam ML Zimmermann M Zarbock A Hönemann C. Improved diagnosis of Iron deficiency Anemia in the critically ill via fluorescence Flowcytometric hemoglobin biomarkers. Cells. (2022) 12:140. doi: 10.3390/cells12010140, PMID: 36611936 PMC9818818

[9] Dalimunthe NN Lubis AR. Usefulness of reticulocyte hemoglobin equivalent in Management of Regular Hemodialysis Patients with Iron deficiency Anemia. Rom J Intern Med. (2016) 54:31–6. doi: 10.1515/rjim-2016-0003, PMID: 27141568

[10] Peerschke EIB Pessin MS Maslak P. Using the hemoglobin content of reticulocytes (RET-he) to evaluate Anemia in patients with Cancer. Am J Clin Pathol. (2014) 142:506–12. doi: 10.1309/AJCPCVZ5B0BOYJGN, PMID: 25239418 PMC4962332

[11] Czempik PF Wiórek A. Comparison of standard and new Iron status biomarkers: a prospective cohort study in Sepsis patients. Healthcare. (2023) 11:995. doi: 10.3390/healthcare11070995, PMID: 37046922 PMC10093794

[12] Bó SD Fragoso ALR Farias MG Hubner DPG de Castro SM. Evaluation of RET-he values as an early indicator of iron deficiency anemia in pregnant women. Hematol Transfus Cell Ther. (2023) 45:52–7. doi: 10.1016/j.htct.2021.05.006, PMID: 34266811 PMC9938494

[13] Villani R Romano AD Rinaldi R Sangineto M Santoliquido M Cassano T. Prevalence and risk factors for hospital-acquired anemia in internal medicine patients: learning from the “less is more” perspective. Intern Emerg Med. (2023) 18:177–83. doi: 10.1007/s11739-022-03147-x, PMID: 36346557 PMC9883305

[14] Thomas J Jensen L Nahirniak S Gibney RTN. Anemia and blood transfusion practices in the critically ill: a prospective cohort review. Heart Lung. (2010) 39:217–25. doi: 10.1016/j.hrtlng.2009.07.002, PMID: 20457342

[15] Warner MA Hanson AC Frank RD Schulte PJ Go RS Storlie CB. Prevalence of and recovery from Anemia following hospitalization for critical illness among adults. JAMA Netw Open. (2020) 3:e2017843. doi: 10.1001/jamanetworkopen.2020.17843, PMID: 32970158 PMC7516623

[16] Apple CG Kelly LS Kannan KB Ungaro RF Moore FA Brakenridge SC. Ineffective erythropoietin response to Anemia in Sepsis. Surg Infect. (2022) 23:142–9. doi: 10.1089/sur.2021.152, PMID: 34958257 PMC8892986

[17] Ginzburg Y An X Rivella S Goldfarb A. Normal and dysregulated crosstalk between iron metabolism and erythropoiesis. eLife. (2023) 12:12. doi: 10.7554/eLife.90189 PMC10425177 37578340

[18] Khalil S Delehanty L Grado S Holy M White Z Freeman K. Iron modulation of erythropoiesis is associated with scribble-mediated control of the erythropoietin receptor. J Exp Med. (2018) 215:661–79. doi: 10.1084/jem.20170396, PMID: 29282252 PMC5789406

[19] Toki Y Ikuta K Kawahara Y Niizeki N Kon M Enomoto M. Reticulocyte hemoglobin equivalent as a potential marker for diagnosis of iron deficiency. Int J Hematol. (2017) 106:116–25. doi: 10.1007/s12185-017-2212-6, PMID: 28299633

[20] Rusch JA van der Westhuizen DJ Gill RS Louw VJ. Diagnosing iron deficiency: controversies and novel metrics. Best Pract Res Clin Anaesthesiol. (2023) 37:451–67. doi: 10.1016/j.bpa.2023.11.001, PMID: 39764832

[21] Danielson K Beshara S Qureshi AR Heimbürger O Lindholm B Hansson M. Delta-he: a novel marker of inflammation predicting mortality and ESA response in peritoneal dialysis patients. Clin Kidney J. (2014) 7:275–81. doi: 10.1093/ckj/sfu038, PMID: 25852889 PMC4377757

[22] Hönemann C Hagemann O Doll D Luedi MM Ruebsam ML Meybohm P. Reticulocyte Haemoglobin as a routine parameter in preoperative Iron deficiency assessment. Endocrinol Metab. (2021) 5:154.

